# Supplementary material for: Genome-Wide Identification, Characterization and Expression Profiling of the CONSTANS-like Genes in Potato (Solanum tuberosum L.)
Source: Genes (Basel). 2023 May 28;14(6):1174. doi: 10.3390/genes14061174 (PMC10297873; doi:10.3390/genes14061174)
Supplement: Supplementary file 1 [file genes-14-01174-s001.zip › Figure S1. COL amino acid sequence alignment of S. tuberosum.pdf]

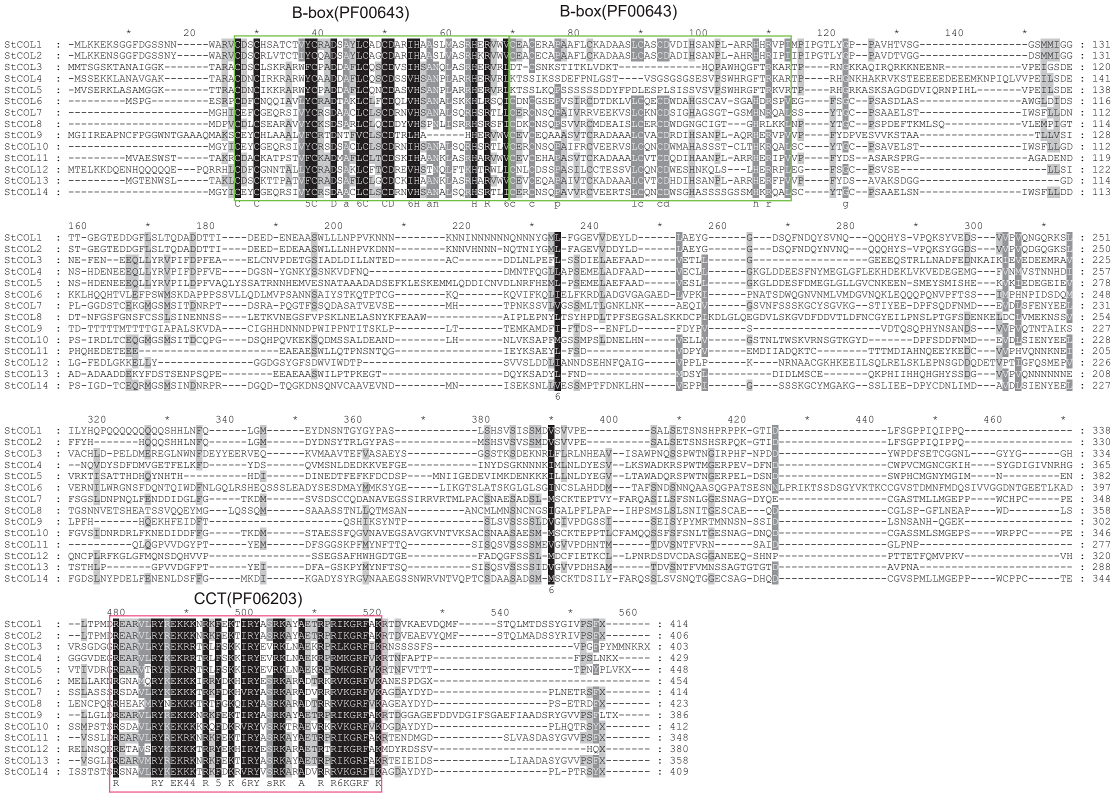

B-box(PF00643)
B-box(PF00643)
CCT(PF06203)
StCOL1
StCOL2
StCOL3
StCOL4
StCOL5
StCOL6
StCOL7
StCOL8
StCOL9
StCOL10
StCOL11
StCOL12
StCOL13
StCOL14
