# Supplementary material for: Genome-Wide Identification, Characterization and Expression Profiling of the CONSTANS-like Genes in Potato (Solanum tuberosum L.)
Source: Genes (Basel). 2023 May 28;14(6):1174. doi: 10.3390/genes14061174 (PMC10297873; doi:10.3390/genes14061174)
Supplement: Supplementary file 1 [file genes-14-01174-s001.zip › Table S1. Information of primer sequence.pdf]

Table S1. Information of primer sequence

Gene	Primer sequence (5′→3′)
StCOL1	ACAGTTGCCATTCCGCAACA
	CAAACCCATACACGCTCGTGG
StCOL2	GCTGGGGAAGTAGTCGATGA
	AGGCACCACACTATCTCCAC
StCOL3	CTGATGACTACTGGCCCGAT
	TCTGTACCGCAATACCCTCG
StCOL4	TCCATTCGGCCAATCCTTTG
	AAATCCACGGTGCCATGATG
StCOL5	TTCATTCGGCGAACCCTTTG
	GGAGTCCGCACCTTTCTAGT
StCOL6	AGCGACATCTGAGAGCAACA
	TTCCCCTGTTCTTTGCCAGA
StCOL7	CCGCTGCAGAACTCTCTACT
	GTCGGTTGTCAGTGATGCTC
StCOL8	AGGCTTTGCTTGCAATGTGA
	TTCTTGAGTCGATGCCCTGT
StCOL9	CCCCAATTGTTTTCCCGGAG
	GTGCGACAGAACACCAGTG
StCOL10	CTTCCAGGGTGTCAATGCTG
	GCAAAGCAAAGGGTAGGAGG
StCOL11	AAATGATCCACACCAGCACG
	ACATACGGATCCACATCGCT
StCOL12	TTGAGGGGTTTAGCGGATGT
	ACTTCCATCACCGCCATACA
StCOL13	TGCAAATCCGTTAGCTCGTC
	TTCAGCTTCTTCCTCCGGTT
StCOL14	CTACCGTGGTGTCAATGCTG
	CGTCTTGCAGCTCATCATGG
EF-1-alpha	GATGGTCAGACCCGTGAACA
	CCTTGGAGTACTTCGGGGTG
