# Supplementary figures and images for: Genome-Wide Identification, Characterization and Expression Profiling of the CONSTANS-like Genes in Potato (Solanum tuberosum L.)
Source: Genes (Basel). 2023 May 28;14(6):1174. doi: 10.3390/genes14061174 (PMC10297873; doi:10.3390/genes14061174)

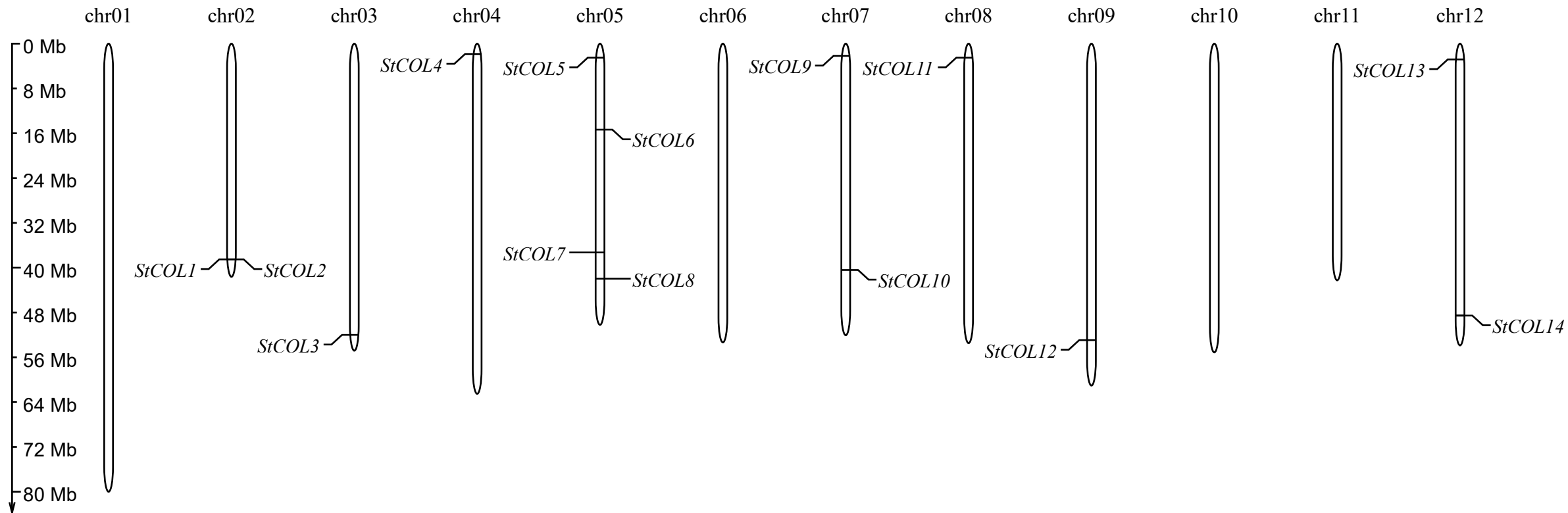

Supplement: Supplementary file 1 [file genes-14-01174-s001.zip › Figure S2. Chromosome distribution of COL genes in S. tuberosum.pdf]

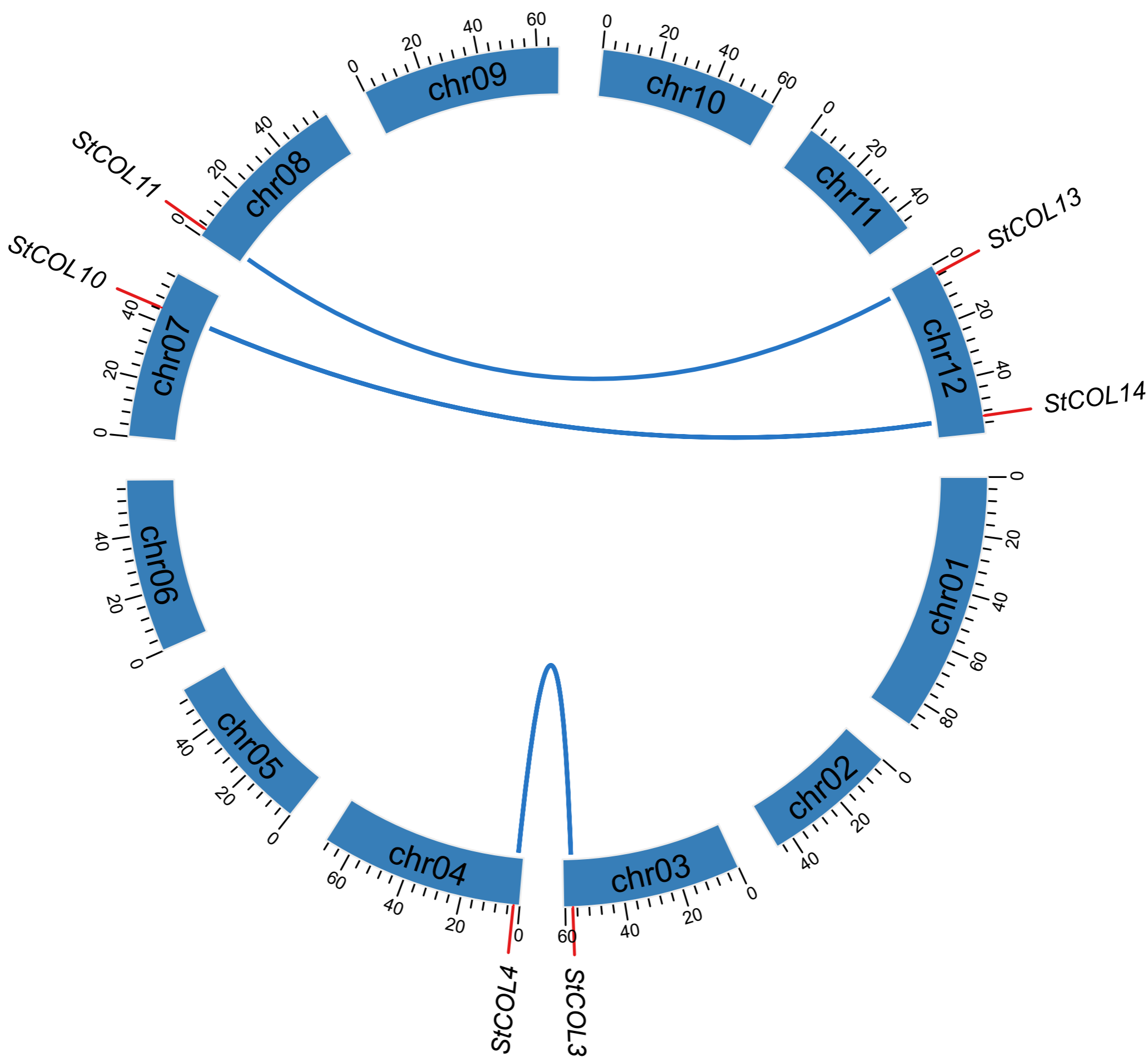

Supplement: Supplementary file 1 [file genes-14-01174-s001.zip › Figure S3. Syntenic relationships of the COL genes within S. tuberosum.pdf]

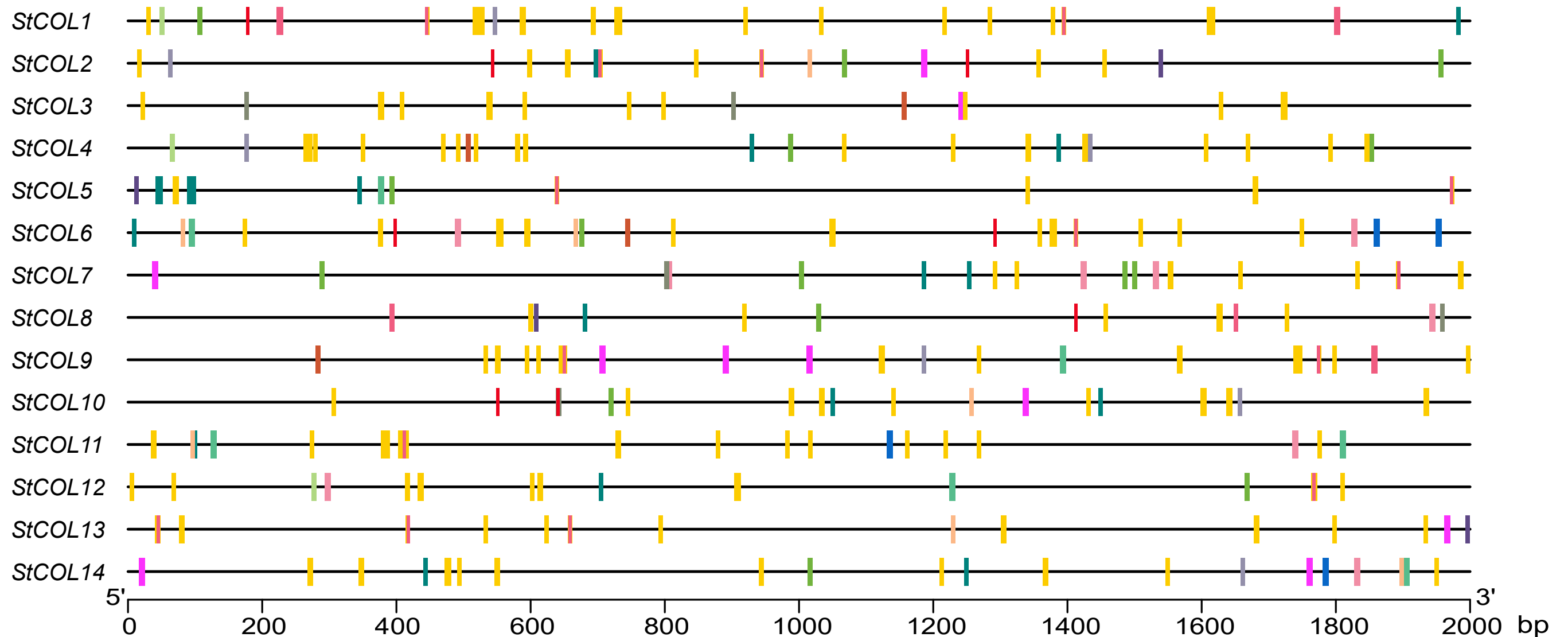

Supplement: Supplementary file 1 [file genes-14-01174-s001.zip › Figure S4. Distribution of cis-elements in the promoter regions of StCOL genes.pdf]
